# Supplementary material for: A call for a unified and multimodal definition of cellular identity in the enteric nervous system
Source: EMBO J. 2025 Sep 15;44(20):5622–39. doi: 10.1038/s44318-025-00559-1 (PMC12528430; doi:10.1038/s44318-025-00559-1)
Supplement: Supplementary file 1 — Appendix [file 44318_2025_559_MOESM1_ESM.pdf]

Appendix for “A call for a unified and multimodal definition of cellular identity in the enteric nervous system”

Table of contents:

Appendix Figure S1: **Page 2**

Appendix Methods: **Page 3**

Appendix Table S1: **Page 3**

Appendix Table S2: **Page 4**

Appendix Figure S1:

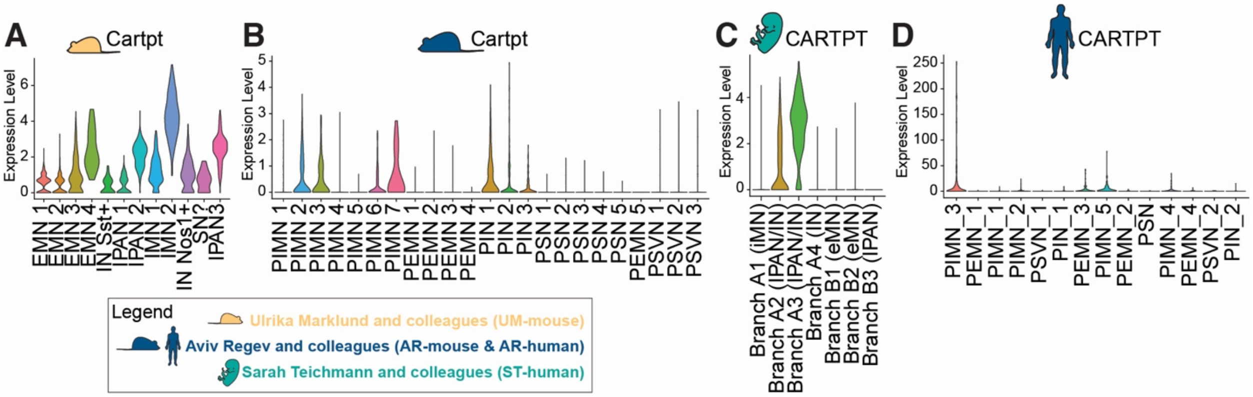

Appendix Figure S1: Expression of Cart peptide gene in primary mouse and human enteric neuron datasets. A) UM-mouse. **B)** AR-mouse. **C)** ST-human. **D)** AR-human

Appendix Methods:

Neurochemical identification of neurons

The neurochemical identification of neurons was performed independently for each neurotransmitter to accommodate multi-neurochemical identities. For each neurotransmitter, a core set of genes were selected consisting of the rate-limiting synthesis enzyme(s), metabolism enzymes and transport proteins (**Appendix Table S1**). Cells were first scored for each neurotransmission associated gene set using the "AddModuleScore" function. A cell was then annotated as "x-ergic" if the cell's expression of a rate limiting enzyme was greater than 0 and the cell's module score for the corresponding gene set was greater than 0. A cell was annotated as "Other" if both criteria were not met. Multi-neurochemical identities were determined by concatenating the individually determined single neurochemical identities of each cell. The overall prevalence of each neurochemical identity per dataset was calculated by summing the total number of cells annotated for each single identity and calculating the percentage of each "x-ergic" identity from this sum total.

Appendix Table S1: Neurochemical ID

Cholinergic	Nitrergic	Dopaminergic	Serotonergic	GABAergic	Glutamatergic
CHAT	NOS1	TH	TPH1	GAD1	GLUL
SLC18A3	NOS1AP	PAH	TPH2	GAD2	SLC17A6
ACHE	ARG1	DDC	DDC	SLC32A1	SLC17A7
SLC5A7	ARG2	TYR	TDO2		SLC17A8
	ASL	SPR	IDO1		
	ASS1	QDPR	IDO2		
		TAT	SLC6A4		
		SLC6A3	SLC18A2		
		QDPR			
		SLC18A2			

Spearman correlations

The transcriptional correlation of between cell clusters in the two datasets was computed using non-imputed gene counts and Seurat's integration functions to first find 100 anchor features based on the first 30 dimensions of the canonical correlation analysis and then integrate the two datasets using the same number of dimensions. The expression of these 100 anchor features was then scaled and centered in the merged data object and the average scaled expression of each anchor feature was calculated for each dataset's cell clusters of interest using the "AverageExpression" function. A Spearman correlation matrix comparing all cell clusters to all cell clusters was generated based on the average scaled expression of the 100 anchor features.

Cell type transcriptional signature module scoring

To find transcriptionally similar neuronal subtypes between two datasets, first the differentially expressed (DE) genes of the reference dataset are calculated from the non-imputed gene counts with the "FindAllMarkers" function using the Wilcoxon Rank Sum test and only genes with a

positive fold change were returned. The DE gene lists are first filtered to remove genes not present in the query dataset. Then for each cell cluster in the reference dataset, a transcriptional signature gene list is made from the top 100 (or as many as possible if <100) DE genes sorted first by LogFC and then increasing adjusted p-value (if two genes has the same LogFC). The query dataset is then scored for the transcriptional signature gene lists of each reference dataset cell cluster using the "AddModuleScore" function based on the query dataset's gene counts (RNA assay, non imputed).

Label transfer

We used SingleCellNet (SCN) (Tan & Cahan, 2019) for unbiased classification of primary enteric neuron subtypes. Reference datasets included studies published by Ulrika Marklund (UM-mouse (Morarach *et al*, 2021)), Aviv Regev (AR-mouse and AR-human (Drokhlyansky *et al*, 2020)), Sarah Teichmann (ST-human(Elmentaite *et al*, 2021)) and colleagues. Feature expression matrices and associated metadata objects were derived from each dataset and used as reference or query datasets. For classification, model training custom parameters were used for each reference dataset. Model was trained on a subset of 100 randomly selected cells for each cell cluster present in the REF reference dataset, selecting top 20 DE genes and top 50 gene pairs for training (**Appendix Table S2**).

Appendix Table S2: SCN assessment parameters

reference-for-query	kappa	AUPRC_w	AUPRC_wc
ST-human for mouse	0.739	0.813	0.893
ST-human for human	0.737	0.827	0.893
AR-human for mouse	0.776	0.875	0.871
AR-human for human	0.779	0.876	0.881
UM-mouse for mouse	0.900	0.929	0.967
UM-mouse for human	0.875	0.913	0.948
AR-mouse for mouse	0.862	0.893	0.942
AR-mouse for human	0.861	0.915	0.942

Harmony integration

Harmony (Korsunsky *et al*, 2019) integration was performed using the Seurat v5 harmony wrapper function. Enteric neuron datasets of the same species were first merged, and metadata was added to track each datasets equivalent of a unique biological sample (termed Dataset_SampleID) by appending a dataset identifier to the existing sample identifier column specific to each dataset (Sample.name for ST-human, Patient_ID for AR-human, Biorep for UM-mouse and Mouse_ID for AR-mouse). Due to the subsetting of each dataset to only include the original author annotated neurons, some samples contributed very few cells to the neuron subset dataset. Samples that consisted of less than 10 cells were removed to comply with the functionality of the subsequent data processing steps. First, the RNA assay was split to create a separate layer for each Dataset_SampleID. Counts normalization and variable feature

identification is then performed on each layer separately to identify a consensus set of variable features across all samples. These shared variable features are then scaled and used for principal component analysis. The individual samples were then integrated using the “IntegrateLayers” function with the method set to Harmony Integration. The nearest neighbor graph construction and UMAP dimensionality reduction were then performed on the first 30 harmony components. Clusters were identified using the Louvain algorithm with a resolution of .3 and .6 for the integrated mouse and human datasets, respectively.

GSEA hierarchical clustering

For each primary ENS dataset, DE genes for each For each primary EN dataset, DE genes for each neuronal cluster were calculated using the “FindAllMarkers” function. Gene set enrichment analysis (GSEA) was performed on each neuronal subytpe’s upregulated DE genes (positive log2 fold change only) sorted by decreasing log2 fold change using fgsea v1.16 for the MSigDB gene ontology (C5) pathways. Normalized Enrichment Scores (NES) were calculated for gene sets containing a minimum of 15 genes in the DE gene list with the scoreType set to “positive”. Recovered enriched pathways were filtered to only include biological process (GOBP) gene sets but not filtered based on significance as to not limit the result to pathways enriched in the highest fold change genes. The NESs of the filtered GSEA results for all clusters were then merged and pathways not detected in a neuronal cluster were assigned a NES of 0. Euclidean distance based hierarchical clustering was then performed based on the NESs to cluster both the gene ontology pathways and the neuronal clusters.

RNA labeling

RNAscope® was performed according to manufacturer’s instructions (RNAscope® Multiplex Fluorescent Reagent Kit v2 Assay) on (15-20) µm FFPE sections of human stomach tissue sections. The following probes were used for the analysis: Hs-SYP (Ref 311421), Hs-CHAT-C2 (Ref 450671-C2) Hs-NOS1-C3 (Ref 506551-C3). Images were acquired using ImageXpress Confocal HT.ai (Molecular Devices).

Preparation of paraffin-embedded human colon sections

Human sigmoid colon tissue was received from the International Institute for the Advancement of Medicine (IIAM) that provides non-transplantable organs from Organ Procurement Organizations for biomedical research purposes. Colon tissue was obtained under sterile conditions, flushed with isotonic solution, submerged in organ transplant solution, and shipped on ice to laboratory within 24 hours post mortem. Full-thickness tissues pieces (~2 cm2) were fixed overnight (<24 hours) in 10% neutral buffered formalin (Cancer Diagnostics, FX1003). Samples were transferred to 70% ethanol prior to paraffin embedding (Leica ASP6025, tissue processor). Approximately 5µM thick transverse tissue sections were cut onto coated glass slides (Superfrost® Plus Micro Slide; VWR, 48311-703) and air-dried overnight. All following slide preparation steps were performed at room temperature. Slides with paraffin sections were washed three times in clean xylene substitute (Sigma A5597), then once each in 100% ethanol, 95% ethanol, and 70% ethanol. Slides were then run under house DI water for 5 minutes before being placed in 1X PBS for storage at 4 °C for up to 4 weeks. Prior to staining, paraffin sections underwent antigen retrieval in either citrate buffer (Vector Laboratories Antigen Unmasking Solution H-3300) or TE buffer

(Thermo 17890, brought to pH 9.0 with 1 M NaOH). Slides were incubated in buffer for 10 minutes at 95 °C using a Pelco BioWave Pro+ set to 400 watts.

Staining enteric ganglioid frozen sections and paraffin-embedded human colon sections
Unless otherwise specified, all steps were performed at room temperature. Paraffin-embedded human normal colon sections were prepared as above and then washed three times in PBS and blocked for 1-2 hours in serum (10% donkey or 10% goat) with 0.5% (v/v) Triton X-100 (VWR 0694). Slides were then incubated with primary antibody diluted in serum (10% donkey or 10% goat) with 0.1% Triton X-100 at 4 °C for 12-20 hours. Slides were washed six times for 20 minutes each in PBS with 0.1% Tween-20 (Sigma P1379) and incubated for 1 hour with Alexa Fluor conjugated secondary antibodies. The diluted secondary antibody solution was removed and replaced with 1.0 μg/mL DAPI in water for 10 minutes. The slides were washed six times for 20 minutes each in PBS with 0.1% Tween-20 and coverslips were mounted with Fluoromount-G (Southern Biotech 0100-01). Images were acquired on a Leica SP8 inverted confocal or on the Echo Revolve.
