# Supplementary material for: A call for a unified and multimodal definition of cellular identity in the enteric nervous system
Source: EMBO J. 2025 Sep 15;44(20):5622–39. doi: 10.1038/s44318-025-00559-1 (PMC12528430; doi:10.1038/s44318-025-00559-1)
Supplement: Supplementary file 4 — Expanded View Figures [file 44318_2025_559_MOESM4_ESM.pdf]

Expanded View Figures

Figure EV1. Expression of cluster-specific markers across primary enteric neuron clusters.

(**A–D**) UMAPs of enteric neurons generated from the original datasets of UM-mouse (**A**), AR-mouse (**B**), AR-human (**C**), and ST-human (**D**). "?" refers to the cluster labeled as "ENC11" or "?" in Morarach et al (Morarach et al, 2021b). (**E–H**) Dot plot of cluster-specific markers originally used for UM-mouse, (**E**) in UM-mouse, (**F**) in AR-mouse, (**G**) in ST-human, (**H**) in AR-human. (**I–L**) Dot plot of cluster-specific markers originally used for ST-human, (**I**) in UM-mouse, (**J**) in AR-mouse, (**K**) in ST-human, (**L**) in AR-human. (**M–P**) Dot plot of cluster-specific markers originally used for AR-mouse and AR-human, (**M**) in UM-mouse, (**N**) in AR-mouse, (**O**) in ST-human, (**P**) in AR-human. (**Q**, **R**) Bar plot proportion of different functional annotations described for (**Q**) NOS1+ and (**R**) PENK+ enteric neurons across primary ENS datasets.

© The Author(s)

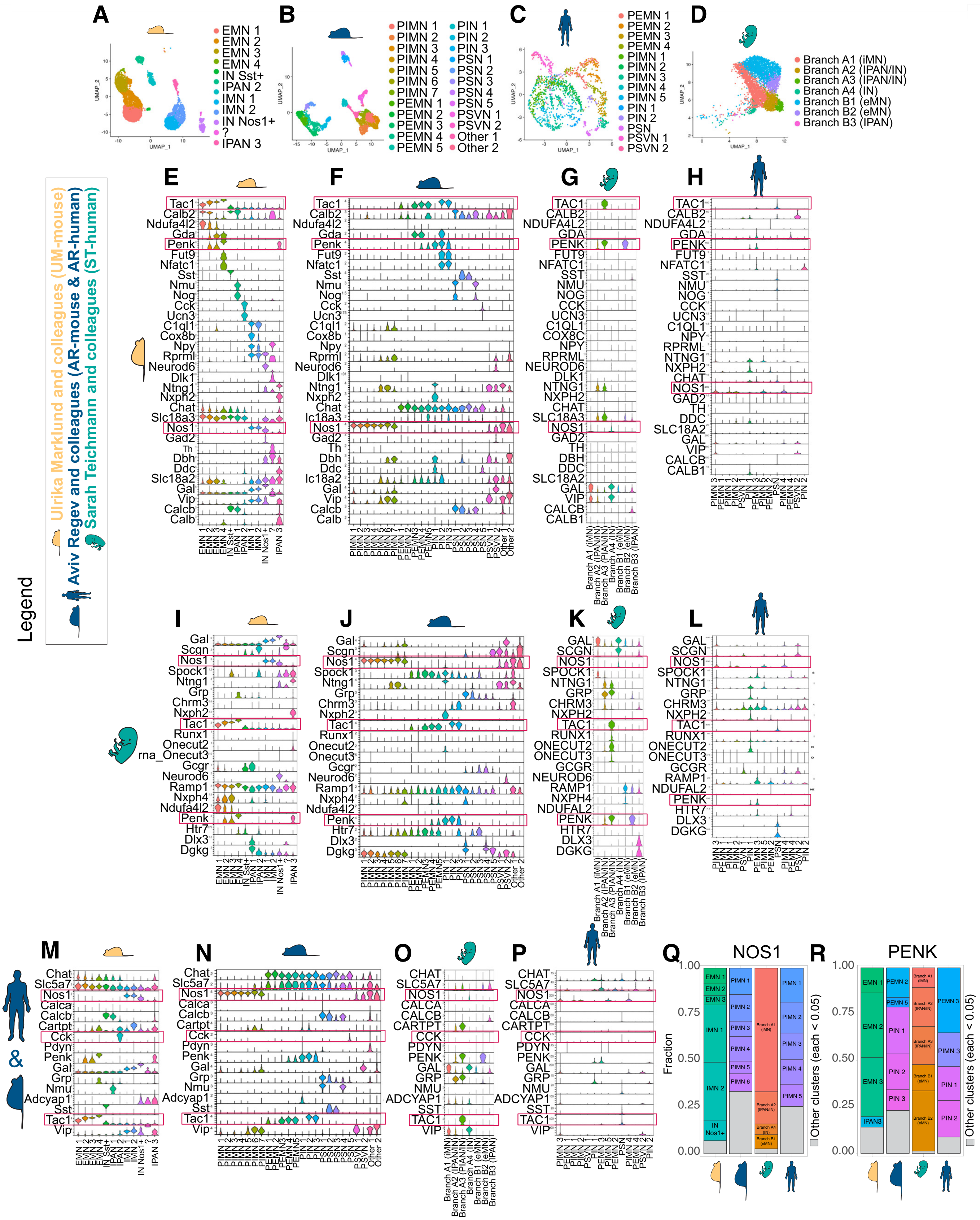
A
B
C
D
E
F
G
H
I
J
K
L
M
N
O
P
Q
R
Legend
Ulrika Marklund and colleagues (UM-mouse)
Aviv Regev and colleagues (AR-mouse & AR-human)
Sarah Teichmann and colleagues (ST-human)
NOS1
PENK
Fraction
Other clusters (each < 0.05)

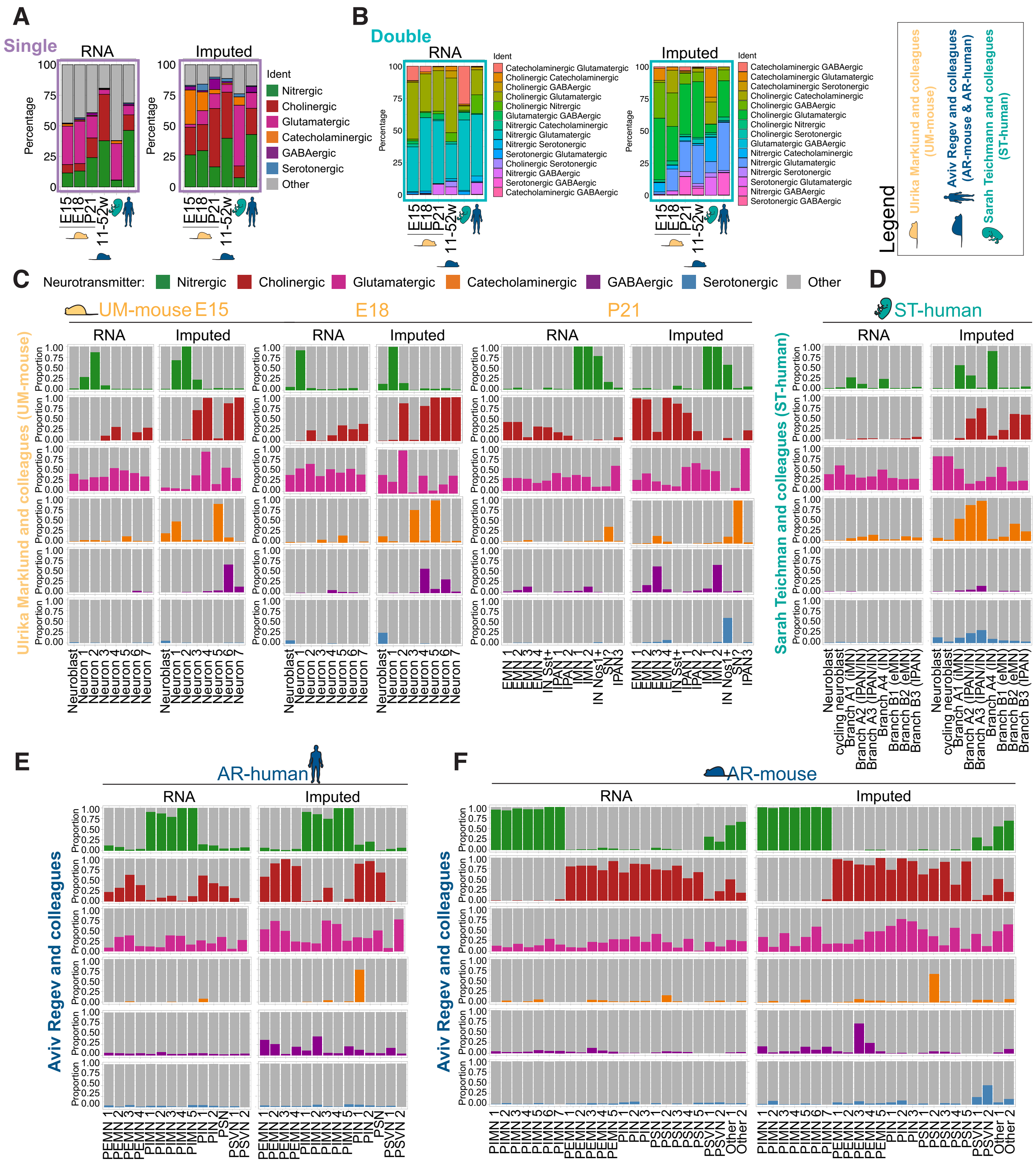

Figure EV2. Neurochemical identities in primary enteric neuron cell types and subtypes.

(**A**) Percentage of single-neurotransmitter-producing enteric neurons in primary datasets. (**B**) Percentage of double-neurotransmitter-producing enteric neurons in primary datasets. (**C–F**) Distribution of neurochemical identities in primary mouse (UM-mouse (**C**), AR-mouse (**F**) and human (ST-human (**D**), AR-human (**E**)) enteric neuron subtypes.

 © The Author(s)

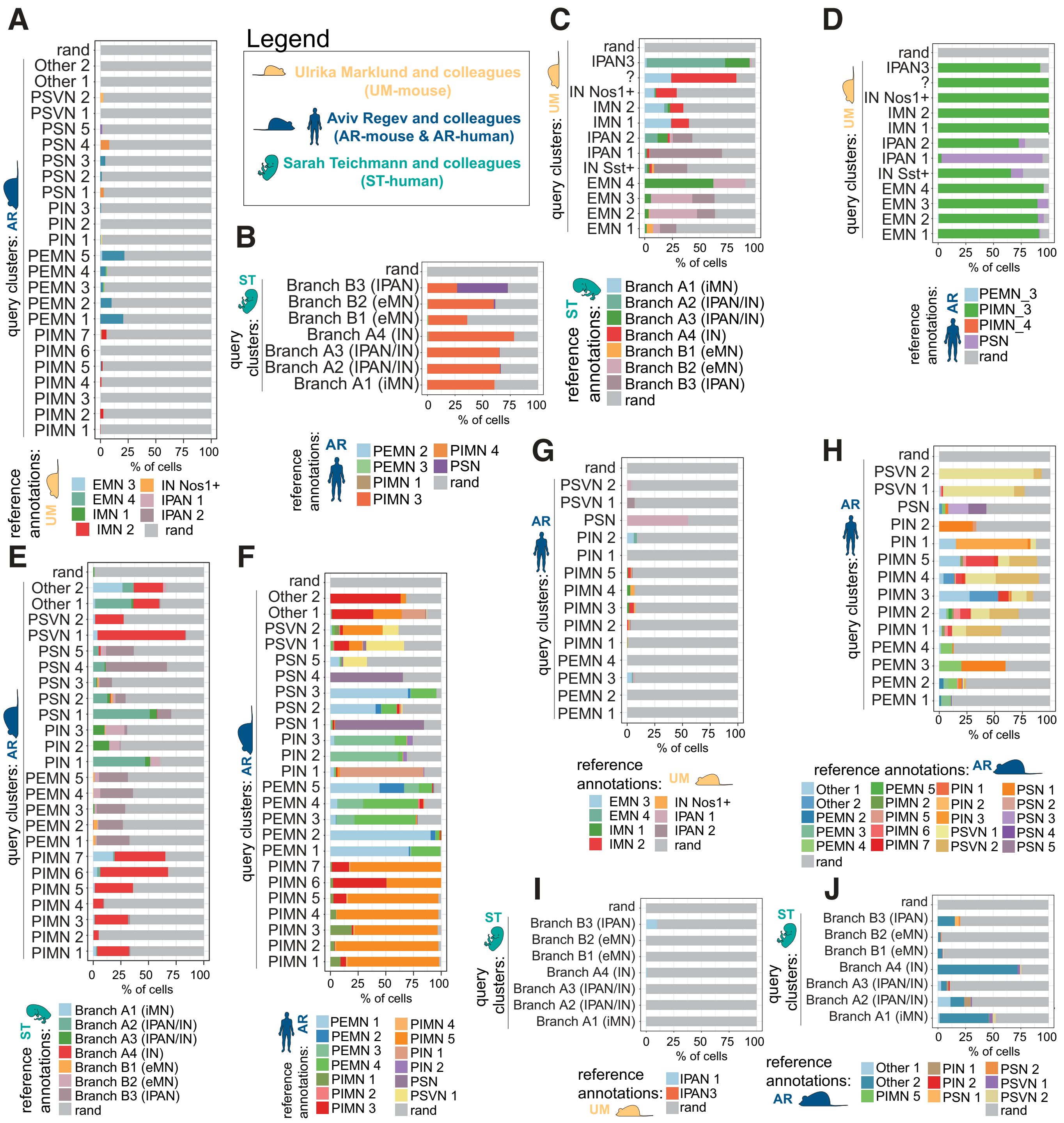

Figure EV3. SingleCellNet unbiased label transfer and classification of primary enteric neurons.

(A–J) Reference primary enteric neuron scRNA-seq datasets of mouse (UM-mouse (**A**, **G**, **I**), AR-mouse (**H**, **J**)) and human (ST-human (**C**, **E**), AR-human (**B**, **D**, **F**)) were used to train SingleCellNet (Tan and Cahan, 2019). These models were then used for label transfer and cross-annotation in the other datasets. Please see Methods for more details.

© The Author(s)

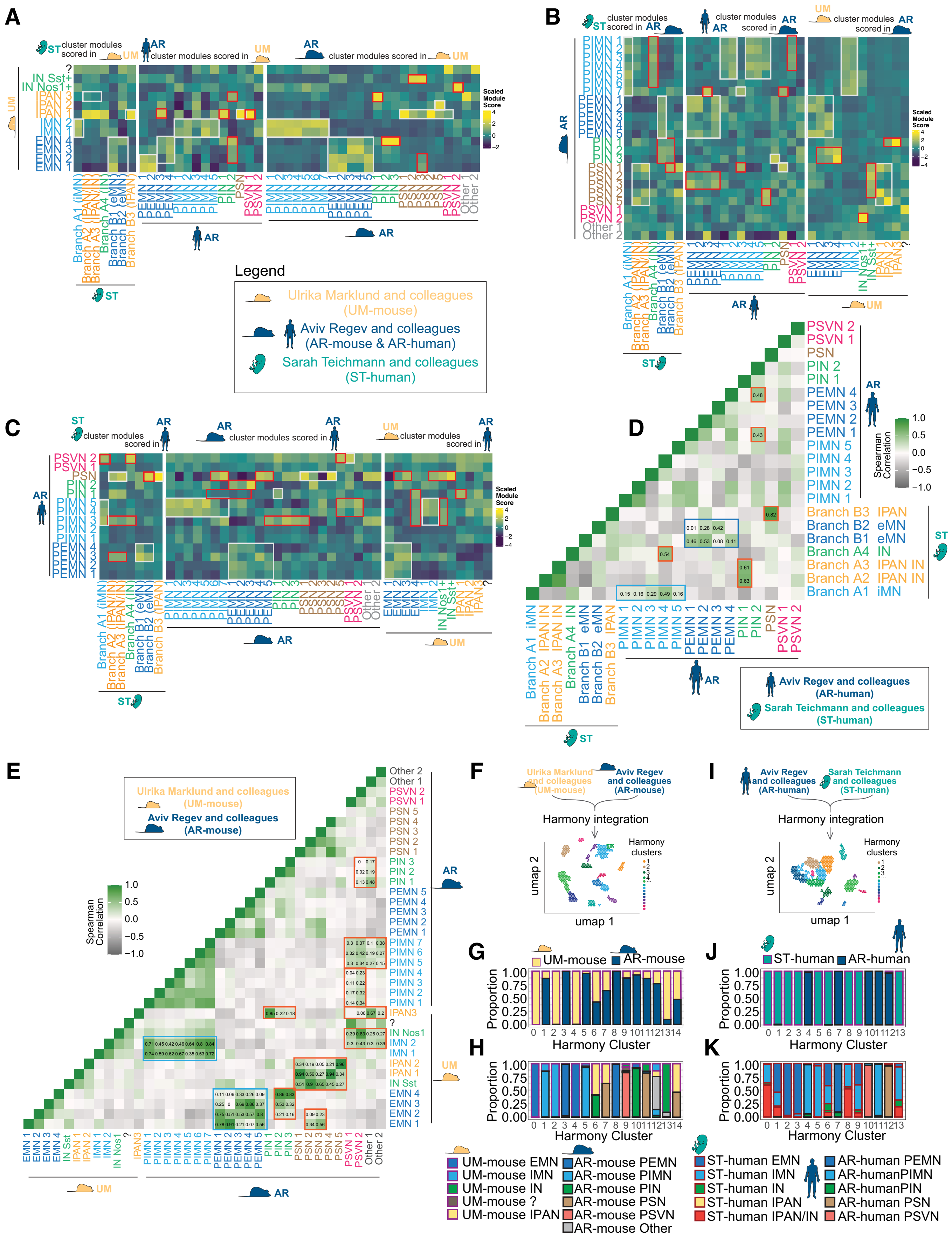
A
cluster modules scored in
ST
UM
AR
Scaled Module Score
Legend
Ulrika Marklund and colleagues (UM-mouse)
Aviv Regev and colleagues (AR-mouse & AR-human)
Sarah Teichmann and colleagues (ST-human)
B
C
D
Spearman Correlation
Aviv Regev and colleagues (AR-human)
Sarah Teichmann and colleagues (ST-human)
E
Ulrika Marklund and colleagues (UM-mouse)
Aviv Regev and colleagues (AR-mouse)
F
Harmony integration
Harmony clusters
umap 1
umap 2
I
G
UM-mouse
AR-mouse
Proportion
Harmony Cluster
H
J
ST-human
AR-human
K
UM-mouse EMN
UM-mouse IMN
UM-mouse IN
UM-mouse ?
UM-mouse IPAN
AR-mouse PEMN
AR-mouse PIMN
AR-mouse PIN
AR-mouse PSN
AR-mouse PSVN
AR-mouse Other
ST-human EMN
ST-human IMN
ST-human IN
ST-human IPAN
ST-human IPAN/IN
AR-human PEMN
AR-humanPIMN
AR-humanPIN
AR-human PSN
AR-human PSVN

 © The Author(s)

◀ **Figure EV4. Cross-dataset module scoring, Spearman correlation of transcriptional signatures and Harmony integration of primary enteric neuron clusters.**

(**A**) Heatmap of the average module scores of ST-human and AR-human neuronal subtype transcriptional signatures in UM-mouse. (**B**) Heatmap of the ST-human, AR-human and UM-mouse neuronal subtype transcriptional signatures in AR-mouse. (**C**) Heatmap of the average module scores of ST-human and UM-mouse neuronal subtype transcriptional signatures in AR-human. (**D**, **E**) Heatmap matrix of Spearman correlations based on expression of 100 anchor features shared significantly variable genes (or anchor features) between (**D**) primary human (ST-human and AR-human) and (**E**) primary mouse (UM-mouse AR-mouse) enteric neuron subtypes. (**F**) Schematic representation of Harmony integration of UM-mouse and AR-mouse datasets. (**G**, **H**) Distribution of cells derived from UM-mouse and AR-mouse datasets (**G**) and their respective broad functional annotations in each Harmony cluster (**H**). (**I**) Schematic representation of Harmony integration of ST-human and AR-human datasets. (**J**, **K**) Distribution of cells derived from ST-human and AR-human datasets (**J**) and their respective broad functional annotations in each Harmony cluster (**K**). Please see Methods for more details.

© The Author(s)

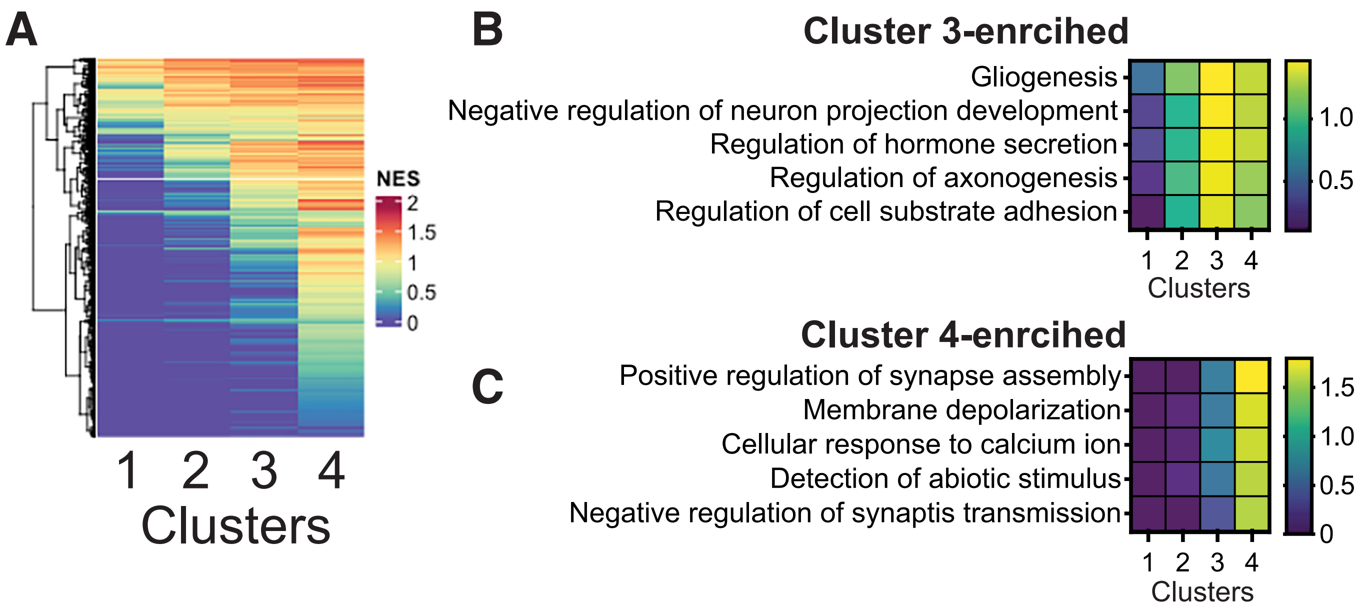

Figure EV5. Hierarchical clustering and comparative analysis of primary ENS neurons.

Hierarchical clustering of primary enteric neuron clusters based on normalized enrichment scores of biological process gene ontology (GOBP) pathways. (**A**) Average enrichment scores of clusters are shown in Fig. 5A. (**B**, **C**) Five representative pathways that show higher enrichment scores in clusters 3 (**B**) and cluster 4 (**C**), respectively. Please see Methods for more details.

 © The Author(s)
